# Genes involved in platelet aggregation and activation are downregulated during acute anaphylaxis in humans

**DOI:** 10.1002/cti2.1435

**Published:** 2022-12-26

**Authors:** Francesca M McGrath, Abbie Francis, Daniel M Fatovich, Stephen PJ Macdonald, Glenn Arendts, Andrew J Woo, Erika Bosio

**Affiliations:** ^1^ Centre for Clinical Research in Emergency Medicine Harry Perkins Institute of Medical Research Perth WA Australia; ^2^ Telethon Kids Institute, Centre for Child Health Research, The University of Western Australia Nedlands WA Australia; ^3^ Discipline of Emergency Medicine, Medical School University of Western Australia Perth WA Australia; ^4^ Emergency Department Royal Perth Hospital Perth WA Australia; ^5^ Emergency Department Fiona Stanley Hospital Perth WA Australia; ^6^ Laboratory for Cancer Medicine Harry Perkins Institute of Medical Research Perth WA Australia; ^7^ School of Medical and Health Sciences Edith Cowan University Perth WA Australia

**Keywords:** allergy, anaphylaxis, microarray, platelet, sepsis, trauma

## Abstract

**Objective:**

Mechanisms underlying the anaphylactic reaction in humans are not fully understood. Here, we aimed at improving our understanding of anaphylaxis by investigating gene expression changes.

**Methods:**

Microarray data set GSE69063 was analysed, describing emergency department (ED) patients with severe anaphylaxis (*n* = 12), moderate anaphylaxis (*n* = 6), sepsis (*n* = 20) and trauma (*n* = 11). Samples were taken at ED presentation (T0) and 1 h later (T1). Healthy controls were age and sex matched to ED patient groups. Gene expression changes were determined using *limma*, and pathway analysis applied. Differentially expressed genes were validated in an independent cohort of anaphylaxis patients (*n* = 31) and matched healthy controls (*n* = 10), using quantitative reverse transcription‐polymerase chain reaction.

**Results:**

Platelet aggregation was dysregulated in severe anaphylaxis at T0, but not in moderate anaphylaxis, sepsis or trauma. Dysregulation was not observed in patients who received adrenaline before T0. Seven genes (*GATA1* (adjusted *P‐*value = 5.57 × 10^−4^), *TLN1* (adjusted *P‐*value = 9.40 × 10^−4^), *GP1BA* (adjusted *P*‐value = 2.15 × 10^−2^), *SELP* (adjusted *P*‐value = 2.29 × 10^−2^), *MPL* (adjusted *P*‐value = 1.20 × 10^−2^), *F13A1* (adjusted *P*‐value = 1.39 × 10^−2^) and *SPARC* (adjusted *P*‐value = 4.06 × 10^−2^)) were significantly downregulated in severe anaphylaxis patients who did not receive adrenaline before ED arrival, compared with healthy controls. One gene (*TLN1* (adjusted *P*‐value = 1.29 × 10^−2^)) was significantly downregulated in moderate anaphylaxis patients who did not receive adrenaline before ED arrival, compared with healthy controls.

**Conclusion:**

Downregulation of genes involved in platelet aggregation and activation is a unique feature of the early anaphylactic reaction not previously reported and may be associated with reaction severity.

## Graphical abstract

We have demonstrated the downregulation of genes involved in platelet aggregation during acute anaphylaxis in humans. Results suggest this response may be limited to patients who do not receive adrenaline before emergency department arrival and may be associated with reaction severity. Downregulation of platelet genes was not observed in patients with sepsis or trauma.

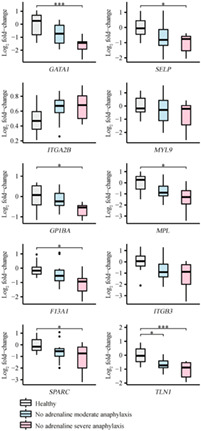

## Introduction

Anaphylaxis is a rapidly developing, potentially life‐threatening reaction that is increasing in incidence worldwide.^1^ Common triggers include drugs, foods and venom stings. Anaphylaxis classically involves IgE‐dependent activation of mast cells, leading to the immediate release of mediators such as histamine, and a peripheral blood cell cascade which results in a systemic reaction.^2^, ^3^ There is now emerging evidence of alternative mechanisms of anaphylaxis, including IgG‐mediated reactions and contact system activation. IgG‐dependent reactions have been demonstrated in mouse models of anaphylaxis and may be responsible for some cases of drug‐triggered reactions in humans.^4^ While mouse models can provide insights into disease mechanisms, they are limited.^5^, ^6^ For example, immunologic disparities exist between mice and humans, including expression of the high affinity IgE receptor, FcεRI, being limited to mast cells and basophils in mice.^5^ Unlike in mice, human platelets express FcεRI and FcεRII, and can be directly activated by IgE.^7^ IgG‐mediated anaphylaxis is thought to be mediated through neutrophil, macrophage, basophil or platelet activation, and release of platelet‐activating factor (PAF), with levels of this mediator correlating with reaction severity.^3^, ^8^, ^9^ The contact system involves a cascade of plasma proteins which drives inflammatory and coagulation pathways.^10^ The contact system can be activated in several ways, including by mast cell‐derived heparin released during IgE‐mediated anaphylaxis.^11^ Numerous factors of the contact system have been shown associated with the severity of anaphylaxis, including levels of plasma heparin, bradykinin formation and intensity of activation.^10^ Additionally, activation of the contact system can contribute towards clinical features of anaphylaxis, including vasodilation, increased vascular permeability, gastro‐intestinal contractions, abdominal pain, angioedema and hypotension.^10^

While our understanding of factors driving the anaphylactic reaction is improving, it is still not fully understood what progresses anaphylaxis into a severe, potentially life‐threatening reaction, and why severity varies between patients. Increasingly, gene expression studies are being used to investigate allergy and anaphylaxis, providing mechanistic insights. An exploratory emergency department (ED) study showed involvement of the innate immune system (particularly neutrophils) and inflammatory response, during acute anaphylaxis in humans.^12^ A recent study on children with food‐induced anaphylaxis demonstrated upregulation of microRNAs (miR‐21‐3p and miR‐487b‐3p) postfood challenge, which are involved in inflammation and immune system regulation.^13^ Gene expression studies utilising mouse models of anaphylaxis have supported human studies, by showing enrichment of genes involved in immune and inflammatory responses, and the complement and coagulation cascades, in the dendritic cells of mice isolated postchallenge.^14^ Other gene expression studies investigating allergy have demonstrated dysregulation of platelet pathways, pathways involved in B‐ and T‐cell development and enrichment of neutrophil and macrophage signatures in allergic individuals.^15^, ^16^, ^17^, ^18^

We have previously interrogated a gene expression data set on anaphylaxis and sepsis, with the aim of identifying dysregulated genes and evaluating biomarker potential.^19^ We reported the upregulation of small nucleolar RNA (snoRNA) networks during acute anaphylaxis, but not sepsis, and highlighted their potential to act as biomarkers.^19^ In the present analysis, we aim to further explore mechanisms driving anaphylaxis, and make additional comparisons with a cohort of trauma patients as an example of noninfectious inflammation in the ED. Overall, comparisons to both sepsis and trauma as examples of acute insults leading to systemic inflammation in ED patients were made to determine whether gene expression changes are unique to anaphylaxis. Further understanding mechanisms and mediators driving anaphylaxis would improve our overall understanding of the anaphylactic reaction, and potentially lead to new therapeutic and diagnostic avenues.

## Results

### Unique biological processes and pathways are associated with severe anaphylaxis in the GSE69063 microarray

Previous analysis on gene set GSE69063 identified significantly dysregulated genes in anaphylaxis patients compared with healthy controls and sepsis patients. Upregulation of a small subset of snoRNAs was validated in an independent cohort.^19^ The focus of this paper was to further interrogate this data set and make additional comparisons with trauma patients as an example of noninfectious inflammation in the ED.

The original analysis on GSE69063 comparing patient groups with healthy controls showed 13, 262 and 2434 downregulated genes at T0, and 12, 460 and 2448 downregulated genes at T1, for moderate anaphylaxis, severe anaphylaxis and sepsis, respectively.^19^ Analysis of the trauma cohort compared with healthy controls showed 874 upregulated genes and 916 downregulated genes at T0, and 1202 upregulated genes and 1131 downregulated genes at T1 (Supplementary figures 1 and 2).

**Figure 1:**
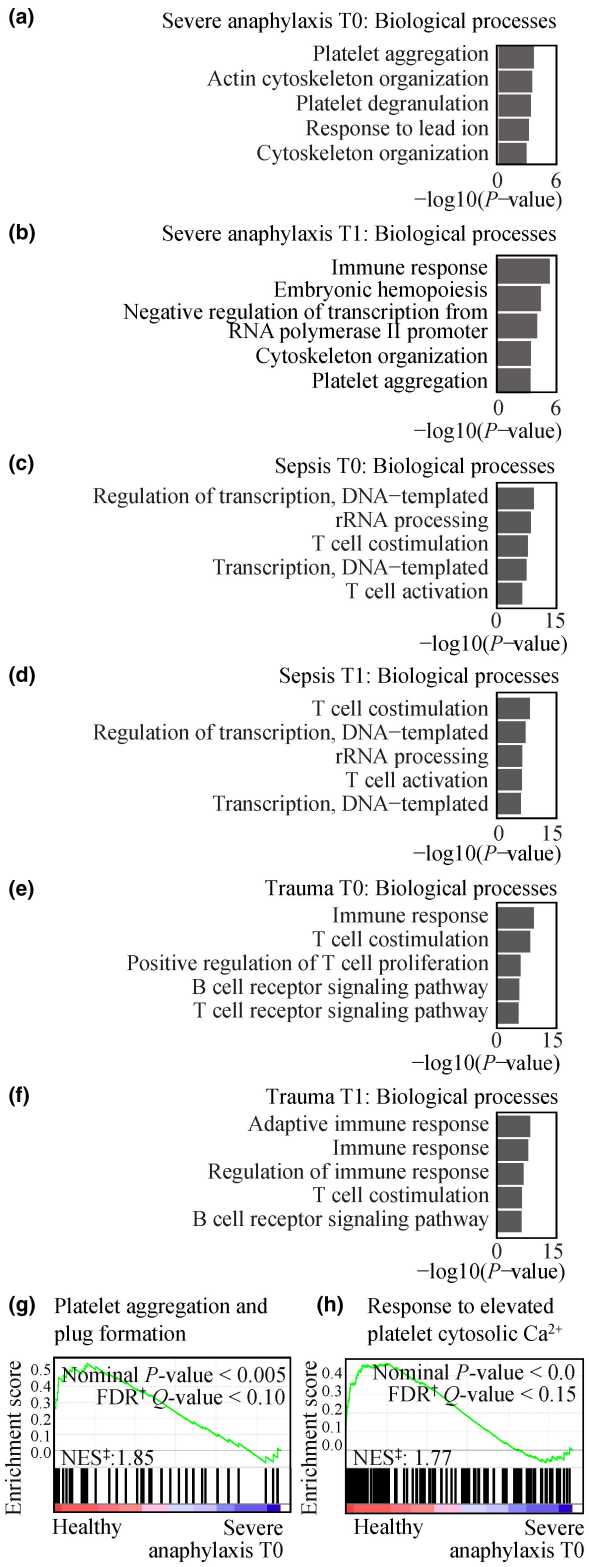
Pathways associated with gene expression in severe anaphylaxis, sepsis and trauma patients, in the GSE69063 microarray cohort. Biological processes associated with downregulated genes (adjusted *P‐*value < 0.05, log fold change ≤ −0.6) in severe anaphylaxis (*n* = 12) at **(a)** T0 and **(b)** T1, sepsis (*n* = 20) at **(c)** T0 and **(d)** T1, and trauma (*n* = 11) at **(e)** T0 and **(f)** T1. Gene Set Enrichment Analysis of the pathways of **(g)** ‘platelet aggregation and plug formation’ and **(h)** ‘response to elevated platelet cytosolic Ca^2+^’ for the comparison of severe anaphylaxis patients and healthy controls, at T0. ^†^FDR, false discovery rate; ^‡^NES, normalised enrichment score.

The Database for Annotation, Visualisation and Integrated Discovery (DAVID) Bioinformatics Resource (version 6.8) gene ontology (GO) and pathway analysis was performed to associate genes downregulated in patients compared to healthy controls with biological processes and pathways. Significant terms associated with the 262 downregulated genes in severe anaphylaxis at T0 included ‘platelet aggregation’ (GO:0070527, *P‐*value = 1.35 × 10^−4^), ‘actin cytoskeleton organisation’ (GO:0030036, *P‐*value = 1.96 × 10^−4^) and ‘platelet degranulation’ (GO:0002576, *P‐*value = 2.69 × 10^−4^; Figure 1a). At T1, the 460 downregulated genes in severe anaphylaxis were associated with ‘immune response’ (GO:0006955, *P‐*value = 1.78 × 10^−6^), ‘embryonic hemopoiesis’ (GO:0035162, *P‐*value = 1.67 × 10^−5^) and ‘negative regulation of transcription from RNA polymerase II promoter’ (GO:0000122, *P‐*value = 4.31 × 10^−5^; Figure 1b). The only significant term associated with the 13 downregulated genes in moderate anaphylaxis at T0 was ‘mesodermal cell differentiation’ (GO:0048333, *P‐*value = 3.27 × 10^−3^). No significant pathways were associated with the 12 downregulated genes in moderate anaphylaxis at T1. Significant terms associated with the 2434 and 2448 downregulated genes in sepsis at T0 and T1, respectively, included ‘regulation of transcription, DNA‐templated’ (GO:0006355, *P‐*value = 8.95 × 10^−11^ and *P‐*value = 1.30 × 10^−8^ for T0 and T1, respectively), ‘rRNA processing’ (GO:0006364, *P‐*value = 5.45 × 10^−10^ and *P‐*value = 9.74 × 10^−8^ for T0 and T1, respectively) and ‘T cell co‐stimulation’ (GO:0031295, *P‐*value = 3.83 × 10^−10^ and *P‐*value = 8.09 × 10^−10^ for T0 and T1, respectively; Figure 1c, d). Significant terms associated with the 916 downregulated genes in trauma at T0 included ‘immune response’ (GO:0006955, *P‐*value = 8.38 × 10^−11^), ‘T cell co‐stimulation’ (GO:0031295, *P‐*value = 8.25 × 10^−10^) and ‘positive regulation of T cell proliferation’ (GO:0042102, *P‐*value = 4.09 × 10^−7^; Figure 1e). Significant terms associated with the 1131 downregulated genes in trauma at T1 include ‘adaptive immune response’ (GO:0002250, *P‐*value = 9.38 × 10^−10^), ‘immune response’ (GO:0006955, *P‐*value = 4.24 × 10^−9^) and ‘regulation of immune response’ (GO:0050776, *P‐*value = 6.69 × 10^−8^; Figure 1f).

Gene Set Enrichment Analysis (GSEA) was performed to confirm dysregulation of platelet pathways shown in severe anaphylaxis patients at T0. The REACTOME pathways of ‘platelet aggregation and plug formation’ and ‘response to elevated platelet cytosolic Ca^2+^’ were found enriched in healthy controls compared with severe anaphylaxis patients at T0 (Figure 1g, h). These pathways were not shown significantly enriched through GSEA analysis in the other patient groups (data not shown).

### Administration of adrenaline before ED arrival results in altered gene pathways

As adrenaline is known to affect platelet activation and aggregation^20^, ^21^, the data (GSE69063) was re‐analysed to compare changes in genes expression in patients with severe anaphylaxis (*n* = 12) on the basis of prehospital administration of adrenaline (*n* = 5), and compared with healthy controls.

**Figure 2:**
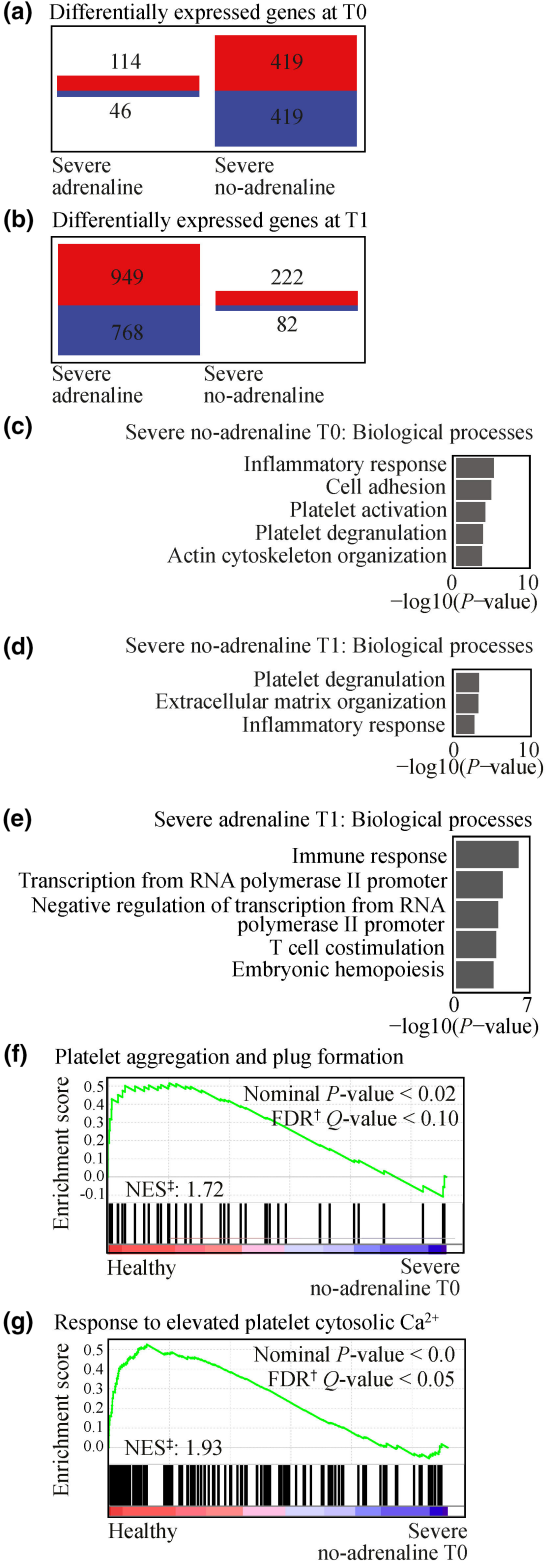
Pathways associated with severe anaphylaxis patients who did or did not receive adrenaline before ED arrival, in the GSE69063 microarray cohort. Numbers of upregulated/downregulated genes (adjusted *P‐*value < 0.05, log fold change ≤ −0.6 or ≥ 0.6) in severe adrenaline (*n* = 5) and severe no‐adrenaline (*n* = 7) groups at **(a)** T0 and **(b)** T1. Biological processes associated with downregulated genes (adjusted *P‐*value < 0.05, log fold change ≤ −0.6) in severe anaphylaxis no‐adrenaline groups at **(c)** T0 and **(d)** T1, and **(e)** severe anaphylaxis adrenaline group at T1. Gene Set Enrichment Analysis of the pathways of **(f)** ‘platelet aggregation and plug formation’ and **(g)** ‘response to elevated platelet cytosolic Ca^2+^’ in the microarray comparison of severe anaphylaxis no‐adrenaline groups to healthy controls at T0. ^†^FDR, false discovery rate. ^‡^NES, normalised enrichment score.

We found 114 and 419 upregulated, and 46 and 419 downregulated genes in severe anaphylaxis patients treated with adrenaline or no adrenaline before ED arrival, respectively, at T0 (Figure 2a). We found 949 and 222 upregulated genes, and 768 and 82 downregulated genes for the severe adrenaline and no‐adrenaline groups at T1 (Figure 2b). DAVID GO analysis found significant terms associated with the 419 downregulated genes in the severe no‐adrenaline group at T0 included ‘inflammatory response’ (GO:0006954, *P‐*value = 4.67 × 10^−6^), ‘cell adhesion’ (GO:0007155, *P‐*value = 1.04 × 10^−5^) and ‘platelet activation’ (GO:0030168, *P‐*value = 7.74 × 10^−5^; Figure 2c). No significant terms were associated with the 46 downregulated genes in the severe adrenaline group at T0. Significant terms associated with the 82 downregulated genes in the severe no‐adrenaline group at T1 included ‘platelet degranulation’ (GO:0002576, *P‐*value = 5.67 × 10^−4^), ‘extracellular matrix organisation’ (GO:0030198, *P‐*value = 7.77 × 10^−4^) and ‘inflammatory response’ (GO:0006954, *P‐*value = 2.69 × 10^−3^; Figure 2d). Significant terms associated with the 768 downregulated genes in the severe adrenaline group at T1 included ‘immune response’ (GO:0006955, *P‐*value = 6.05 × 10^−7^), ‘transcription from RNA polymerase II promoter’ (GO:0006366, *P‐*value = 2.39 × 10^−5^) and ‘negative regulation of transcription from RNA polymerase II promoter’ (GO:0000122, *P‐*value = 6.76 × 10^−5^; Figure 2e).

At T0, GSEA showed that REACTOME pathways of ‘platelet aggregation and plug formation’ and ‘response to elevated platelet cytosolic Ca^2+^^’^ were enriched in healthy controls compared with severe no‐adrenaline patients (Figure 2f, g). Dysregulation of these pathways was not seen in severe adrenaline groups at either time point, or the severe no‐adrenaline group at T1 (data not shown).

### qRT‐PCR analysis validates downregulation of platelet genes

An independent cohort of 31 anaphylaxis (12 severe, 22 no‐adrenaline) patients and 10 age‐ and sex‐matched healthy controls were selected for validation of the GSE69063 microarray. Clinical data characterising the anaphylaxis patients in the validation cohort are described in Table 1. Timing between anaphylaxis onset and blood sampling ranged from 17 min to 2 h and 30 min (median 65 min). Anaphylaxis was primarily triggered by drugs (*n* = 11, 35%), food (*n* = 9, 29%) and venom (bee sting, *n* = 6, 19%). All cases of severe anaphylaxis presented with cardiovascular features, as was seen in the microarray cohort (Table 1). While in the microarray cohort, the majority of severe reactions were caused by drugs (83%), and all moderate reactions were caused by foods, in the validation cohort 50% of severe cases were caused by drugs, and only 37% of moderate reactions were caused by foods.

**Table 1:** Clinical reaction features of trauma, sepsis and anaphylaxis presentations assessed in the microarray and validation cohorts.

	*Trauma patients*		*Sepsis patients*	
	Trauma microarray	Trauma validation	Sepsis microarray	Sepsis validation
*n*	11	20	20	20
Age, mean (SD)	38.6 (21.4)	43.8 (21.2)	58.20 (16.1)	59.25 (19.6)
Male sex, *n* (%)	9.0 (82.0)	13.0 (65.0)	10 (50)	13 (65)
MAP (mm Hg) (median) (IQR)	96.0 (32.0)	82.5 (36.8)	88.50 (32)	56.50 (16.3)
WCC (x 10^9^ L^−1^) (median) (IQR)	13.4 (4.7)	15.5 (6.7)	10.6 (7.1)	10.70 (5.8)
Lactate (mmol L^−1^) (median) (IQR)	1.9 (1.3)	3.1 (3.4)	2.55 (2.4)	1.75 (1.2)
CCI (median) (IQR)	0.0 (1.0)	0.0 (1.0)	1 (2.5)	2.50 (3)
Med score (median) (IQR)	2.0 (1.0)	3.0 (1.0)	‐	‐
SOFA score (median) (IQR)	‐	‐	4 (7)	7 (3)
Hypoxia (blood oxygen ≤ 92%), *n* (%)	‐	2 (10)	‐	2 (10)
Hypotension (SBP^††^ < 90 mm Hg), *n* (%)	‐	2 (10)	2 (10)	11 (55)
Length of stay (days) (median) (IQR)	11.0 (29.2)	17.0 (24.7)	7.7 (11.4)	7.50 (11.6)
Death within 30 days, *n* (%)	5.0 (45.0)	8.0 (40.0)	4 (20)	4 (20)

	Anaphylaxis microarray		Anaphylaxis validation cohort	
	Severe anaphylaxis	Moderate anaphylaxis	Severe anaphylaxis	Moderate anaphylaxis
*n*	12	6	12	19
Age, mean (SD) (years)	45.8 (10.8)	50.6 (19.0)	37.2 (11.1)	35.1 (16.1)
Male sex, *n* (%)	3 (25.0)	1 (16.7)	7 (58.3)	10 (52.6)
Suspected cause	‐	‐		
Food, *n* (%)	2 (16.7)	6 (100.0)	2 (16.7)	7 (36.8)
Non‐steroidal anti‐inflammatory drugs, *n* (%)	3 (25.0)	‐	3 (25.0)	3 (15.8)
Beta‐lactam antibiotics, *n* (%)	7 (58.3)	‐	‐	2 (10.5)
Drug other/uncertain, *n* (%)	‐	‐	3 (25.0)	‐
Venoms, *n* (%)	‐	‐	3 (25.0)	3 (15.8)
Physical, *n* (%)	‐	‐	1 (8.3)	1 (5.3)
Pollen, *n* (%)	‐	‐	‐	1 (5.3)
Unknown, *n* (%)	‐	‐	‐	2 (10.5)
Onset to first sample, median (IQR) (min)	62.5 (63)	63.5 (33.8)	57.50 (35.8)	72 (28.5)
Any skin features, *n* (%)	11 (91.7)	5 (83.3)	12 (100.0)	19 (100.0)
Urticaria, *n* (%)	6 (50.0)	1 (16.7)	9 (75.0)	9 (47.4)
Erythema, *n* (%)	9 (75.0)	2 (33.3)	7 (58.3)	14 (73.7)
Angioedema, *n* (%)	5 (41.7)	3 (50.0)	4 (33.3)	6 (31.6)
Periorbital oedema, *n* (%)	4 (33)	1 (16.7)	2 (16.7)	3 (15.8)
Any gastrointestinal features, *n* (%)	5 (41.7)	4 (66.7)	4 (33.3)	5 (26.3)
Nausea, *n* (%)	5 (41.7)	3 (50.0)	4 (33.3)	5 (26.3)
Vomiting, *n* (%)	2 (16.7)	1 (16.7)	2 (16.7)	3 (15.8)
Abdominal/pelvic pain, *n* (%)	2 (16.7)	1 (16.7)	‐	3 (15.8)
Any respiratory features, *n* (%)	10 (83.3)	6 (100.0)	12 (100.0)	18 (94.7)
Chest/throat tightness, *n* (%)	7 (58.3)	5 (83.3)	7 (58.3)	13 (68.4)
Dyspnoea, *n* (%)	7 (58.3)	4 (66.7)	6 (50.0)	8 (42.1)
Stridor, *n* (%)	1 (8.3)	1 (16.7)	1 (8.3)	2 (10.5)
Wheeze, *n* (%)	4 (33.3)	3 (50.0)	4 (33.3)	8 (42.1)
Any cardiovascular features, *n* (%)	12 (100.0)	1 (16.7)	12 (100.0)	2 (10.5)
Dizziness, *n* (%)	‐	‐	2 (16.7)	2 (10.5)
Diaphoresis, *n* (%)	4 (33.3)	1 (16.7)	1 (8.3)	‐
Hypotension (SBP < 90 mm Hg), *n* (%)	10 (83.3)	‐	7 (58.3)	‐
Confusion, *n* (%)	3 (25.0)	‐	1 (8.3)	‐
Collapse, *n* (%)	4 (33.3)	‐	7 (58.3)	‐
Loss of consciousness, *n* (%)	2 (16.7)	‐	‐	‐
Cyanosis, (blood oxygen ≤ 92%), *n* (%)	3 (25.0)	‐	1 (8.3)	‐
Treated with adrenaline, *n* (%)	12 (100.0)	6 (100.0)	11 (91.7)	14 (73.7)
Prior to ED only, *n* (%)	1 (8.3)	‐	2 (16.7)	2 (10.5)
In ED only, *n* (%)	7 (58.3)	5 (83.3)	6 (50.0)	10 (52.6)
Both prior to and in ED, *n* (%)	4 (33.3)	1 (16.7)	3 (15.8)	2 (10.5)
Length of stay, median (IQR) (days)	0.55 (0.53)	0.25 (0.33)	0.6 (0.7)	0.20 (0.4)
Death, *n* (%)	‐	‐	‐	‐

CCI, Charlson comorbidity index; ED, emergency department. Data included in this table under ‘sepsis patients’ and ‘anaphylaxis microarray’ have been previously published^19^; IQR, interquartile range; MAP, mean arterial pressure; SBP, systolic blood pressure; SD, standard deviation; SOFA, sequential organ failure assessment; WCC, total White blood Cell Count.

The focus of this validation was to confirm the dysregulation of genes involved in the pathways of platelet aggregation and degranulation demonstrated in the microarray comparison of severe anaphylaxis and healthy controls in gene set GSE69063. A group of 10 genes – *GATA1*, *SELP*, *ITGA2B*, *MYL9*, *GP1BA*, *MPL*, *F13A1*, *ITGB3*, *SPARC* and *TLN1* – were chosen for validation and will subsequently be referred to as the ‘validation panel’ (Table 2). These genes were chosen based on microarray analysis of severe anaphylaxis compared with healthy controls at T0 (adjusted *P‐*value < 0.05), and gene involvement in the DAVID GO pathways of platelet aggregation or degranulation, and/or core enrichment in the GSEA analysis of the REACTOME pathway of platelet aggregation and plug formation at T0 (Table 2).

**Table 2:** Validation panel genes, selected based on significance in microarray analysis, GSEA, or DAVID pathway analysis in severe anaphylaxis at T0 compared with healthy controls.

Gene name	Microarray (adjusted *P*‐value)^a^	GSEA^b^	DAVID^c^
GATA1	2.87 × 10^−2^	NO	YES
SELP	1.52 × 10^−3^	NO	YES
ITGA2B	3.44 × 10^−2^	YES	YES
MYL9	1.02 × 10^−2^	NO	YES
GP1BA	1.09 × 10^−3^	YES	YES
MPL	4.23 × 10^−3^	YES	YES
F13A1	1.25 × 10^−3^	NO	YES
ITGB3	3.86 × 10^−3^	YES	YES
SPARC	5.12 × 10^−3^	NO	YES
TLN1	3.85 × 10^−2^	YES	NO

Genes chosen for validation based on:

^a^ The microarray analysis comparison of severe anaphylaxis groups to healthy controls (adjusted *P‐*value < 0.05).

^b^ Core enrichment in healthy controls compared with severe anaphylaxis at T0 in Gene Set Enrichment Analysis (GSEA) of the RECTOME pathway of platelet aggregation and plug formation.

^c^ Gene involvement in the Database for Annotation, Visualisation and Integrated Discovery (DAVID) biological process of platelet aggregation or platelet degranulation (the Benjamini–Hochberg false discovery rate *q* < 0.50 and *P‐*value < 0.01).

**Figure 3:**
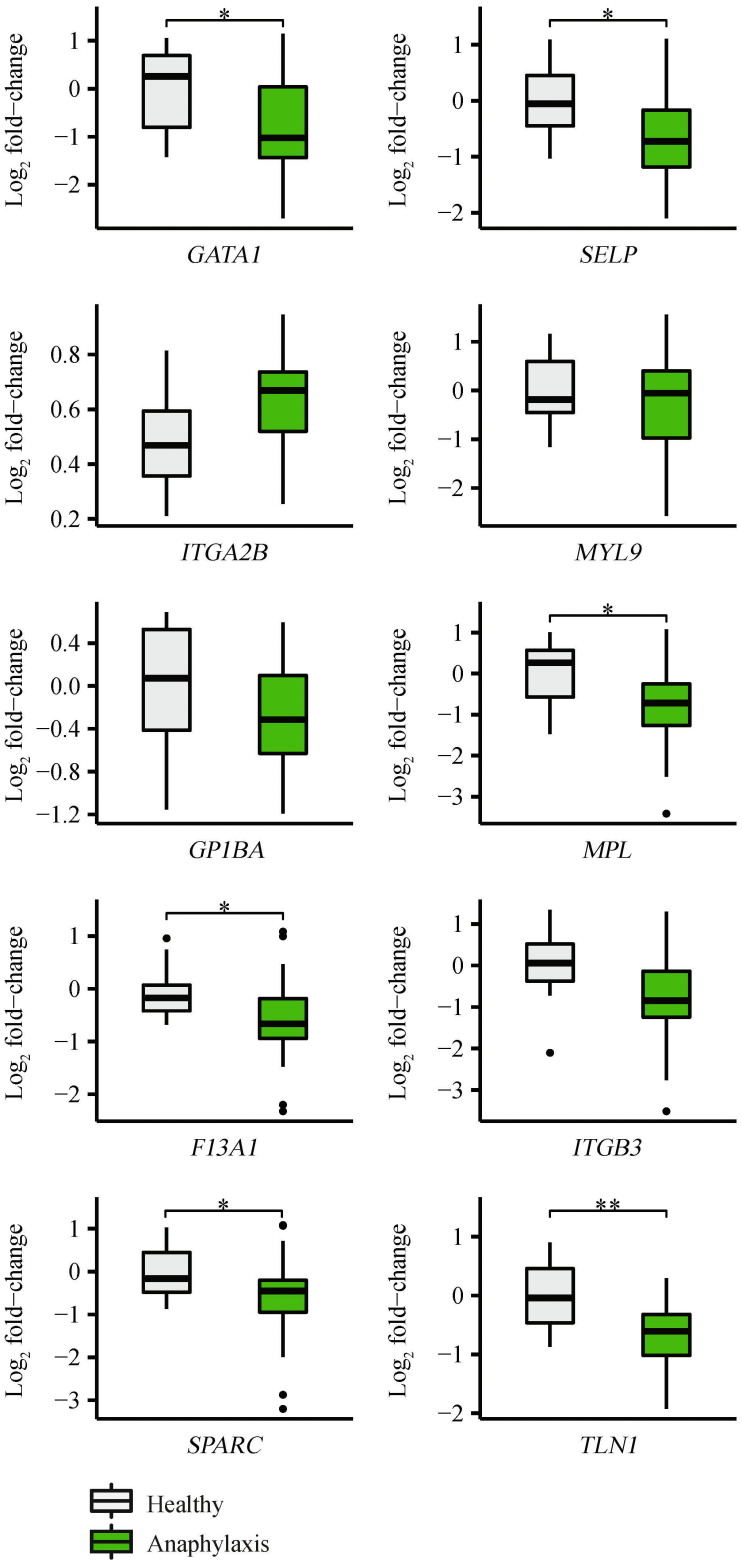
qRT‐PCR validation of platelet‐related validation panel genes for the comparison of anaphylaxis patients (*n* = 31) to healthy controls (*n* = 10). Log_2_ fold‐changes were determined using the 2^−ΔΔCT^ method where groups were normalised to healthy controls. Welch's unequal variance *t*‐tests were used to determine statistically significant differences between experiment groups. *adjusted *P‐*value < 0.05, **adjusted *P‐*value < 0.01.

**Figure 4:**
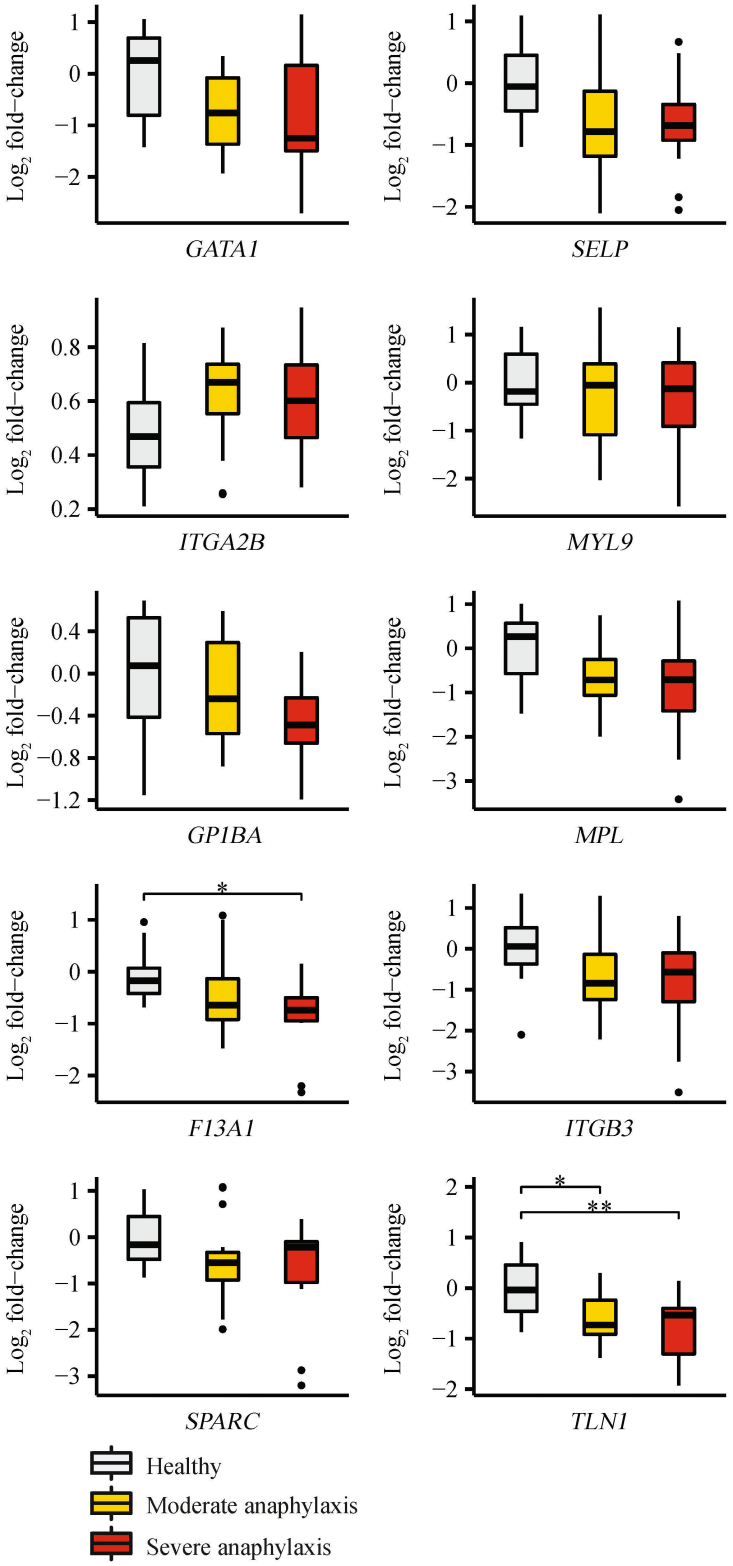
qRT‐PCR validation of platelet‐related validation panel genes for anaphylaxis patients grouped by severity. Log_2_ fold changes were determined using the 2^−ΔΔCT^ method where groups were normalised to healthy controls. Comparison groups included severe anaphylaxis (*n* = 12), moderate anaphylaxis (*n* = 19) and healthy controls (*n* = 10). One‐way ANOVA with the Tukey's honestly significant difference (HSD) adjustment, or the Kruskal–Wallis test with the Bonferroni adjustment, were used to determine statistically significant differences between experiment groups. *adjusted *P‐*value < 0.05, **adjusted *P‐*value < 0.01.

An initial analysis was performed comparing all anaphylaxis patients (*n* = 31) to healthy controls (*n* = 10). Six genes (*GATA1* (*P‐*value = 2.52 × 10^−2^), *SELP* (*P‐*value = 1.86 × 10^−2^), *MPL* (*P‐*value = 2.72 × 10^−2^), *F13A1* (*P‐*value = 1.62 × 10^−2^), *SPARC* (*P‐*value = 2.97 × 10^−2^) and *TLN1* (*P‐*value = 6.13 × 10^−3^)) were significantly downregulated in anaphylaxis patients compared with healthy controls (Figure 3). Subsequent analysis compared anaphylaxis patients grouped by severity (severe (*n* = 12) or moderate (*n* = 19)), with healthy controls (*n* = 10). Two genes (*TLN1* (adjusted *P‐*value = 5.47 × 10^−3^) and *F13A1* (adjusted *P‐*value = 3.32 × 10^−2^)) were significantly downregulated in severe anaphylaxis compared with healthy controls (Figure 4). *TLN1* was also significantly downregulated in moderate anaphylaxis compared with healthy controls (adjusted *P‐*value = 2.19 × 10^−2^, Figure 4). No genes were significantly dysregulated between the moderate and severe groups.

**Figure 5:**
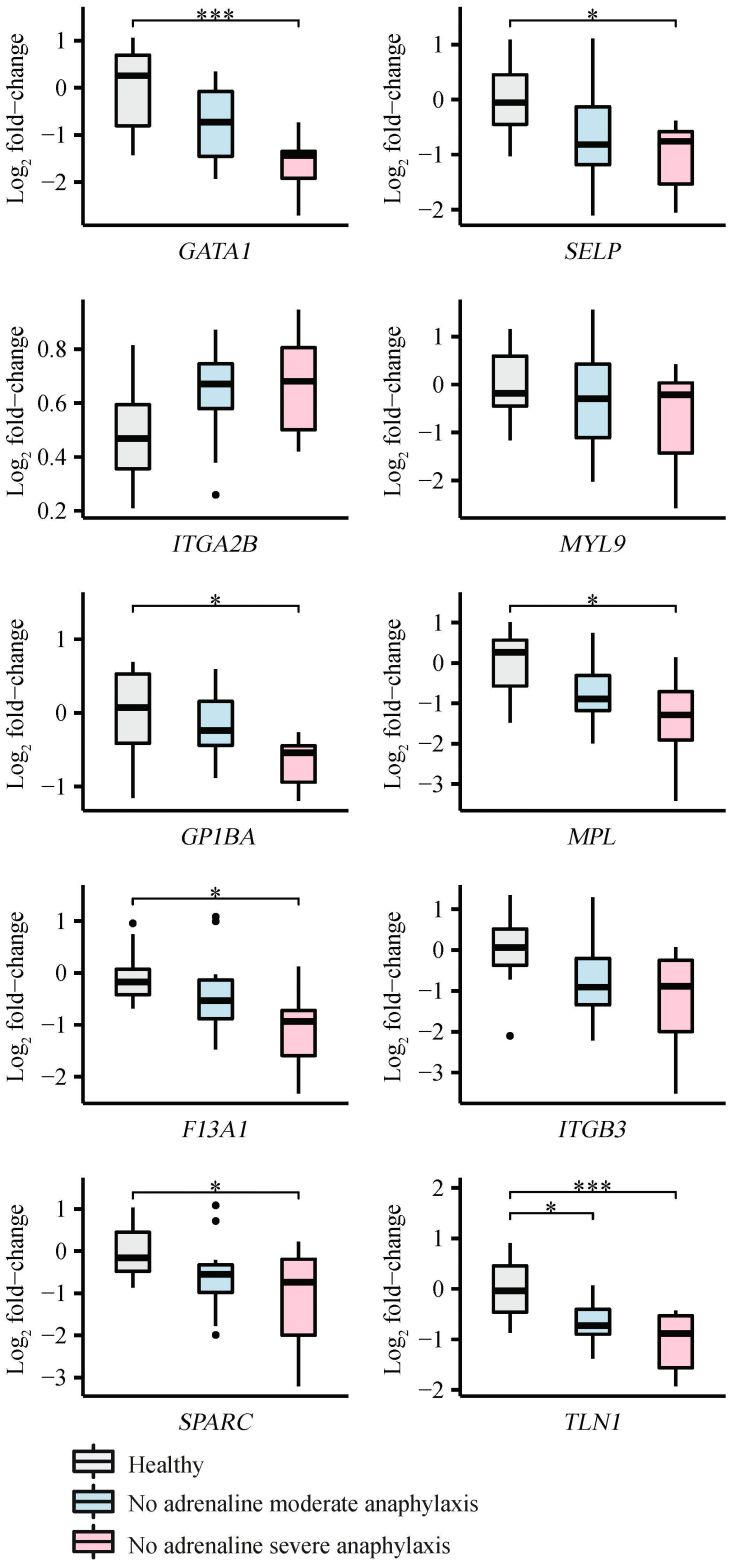
qRT‐PCR validation of platelet‐related validation panel genes for anaphylaxis patients who did not receive adrenaline before ED arrival, grouped by severity. Log_2_ fold‐changes were determined using the 2^−ΔΔCT^ method where groups were normalised to healthy controls. Comparison groups included anaphylaxis patients who did not receive adrenaline before ED arrival (*n* = 22), grouped as severe (*n* = 7), or moderate (*n* = 15), and healthy controls (*n* = 10). One‐way ANOVA with the Tukey's honestly significant difference (HSD) adjustment was used to determine statistically significant differences between experiment groups. *adjusted *P‐*value < 0.05, ***adjusted *P‐*value < 0.001.

Given the effect of adrenaline on gene expression previously noted, gene expression patterns were explored in this validation cohort considering anaphylaxis severity as well as treatment with adrenaline, and compared with healthy controls. The no‐adrenaline and adrenaline anaphylaxis groups consisted of 22 patients (seven severe, 15 moderate) and nine patients (five severe, four moderate), respectively. We found seven genes (*GATA1* (adjusted *P‐*value = 5.57 × 10^−4^), *SELP* (adjusted *P‐*value = 2.29 × 10^−2^), *GP1BA* (adjusted *P‐*value = 2.15 × 10^−2^), *MPL* (adjusted *P‐*value = 1.20 × 10^−2^), *F13A1* (adjusted *P‐*value = 1.39 × 10^−2^), *TLN1* (adjusted *P‐*value = 9.40 × 10^−4^) and *SPARC* (adjusted *P*‐value = 4.06 × 10^−2^)) significantly downregulated in the severe anaphylaxis patients who did not receive adrenaline before ED arrival, compared with healthy controls (Figure 5). One gene (*TLN1* (adjusted *P‐*value = 1.29 × 10^−2^)) was also significantly downregulated in moderate anaphylaxis patients who did not receive adrenaline before ED arrival compared with healthy controls (Figure 5). No genes were significantly dysregulated between severe and moderate no‐adrenaline groups. No genes were significantly downregulated in patients who received adrenaline before ED arrival (severe or moderate) compared with healthy controls (Supplementary figure 3).

### Platelet counts of anaphylaxis patients are within the healthy range

To further understand the involvement of platelets in anaphylaxis, platelet counts of anaphylaxis, sepsis and trauma groups were compared. A healthy baseline of 150–450 × 10^9^ platelets L^−1^ was also used for comparison.^22^, ^23^ The aim was to determine whether platelet numbers were altered during acute anaphylaxis, and whether this was a unique feature of the anaphylactic reaction. A combination of patients from the microarray GSE69063 cohort, anaphylaxis validation cohort and additional sepsis and trauma patients (sepsis and trauma validation cohorts) were used for analysis. Combined, the cohort included 100 patients (29 anaphylaxis (17 severe, 20 no‐adrenaline), 40 sepsis and 31 trauma patients). Clinical characteristics of the sepsis validation cohort (*n* = 20) and trauma validation cohort (*n* = 20) can be found in Table 1.

**Figure 6:**
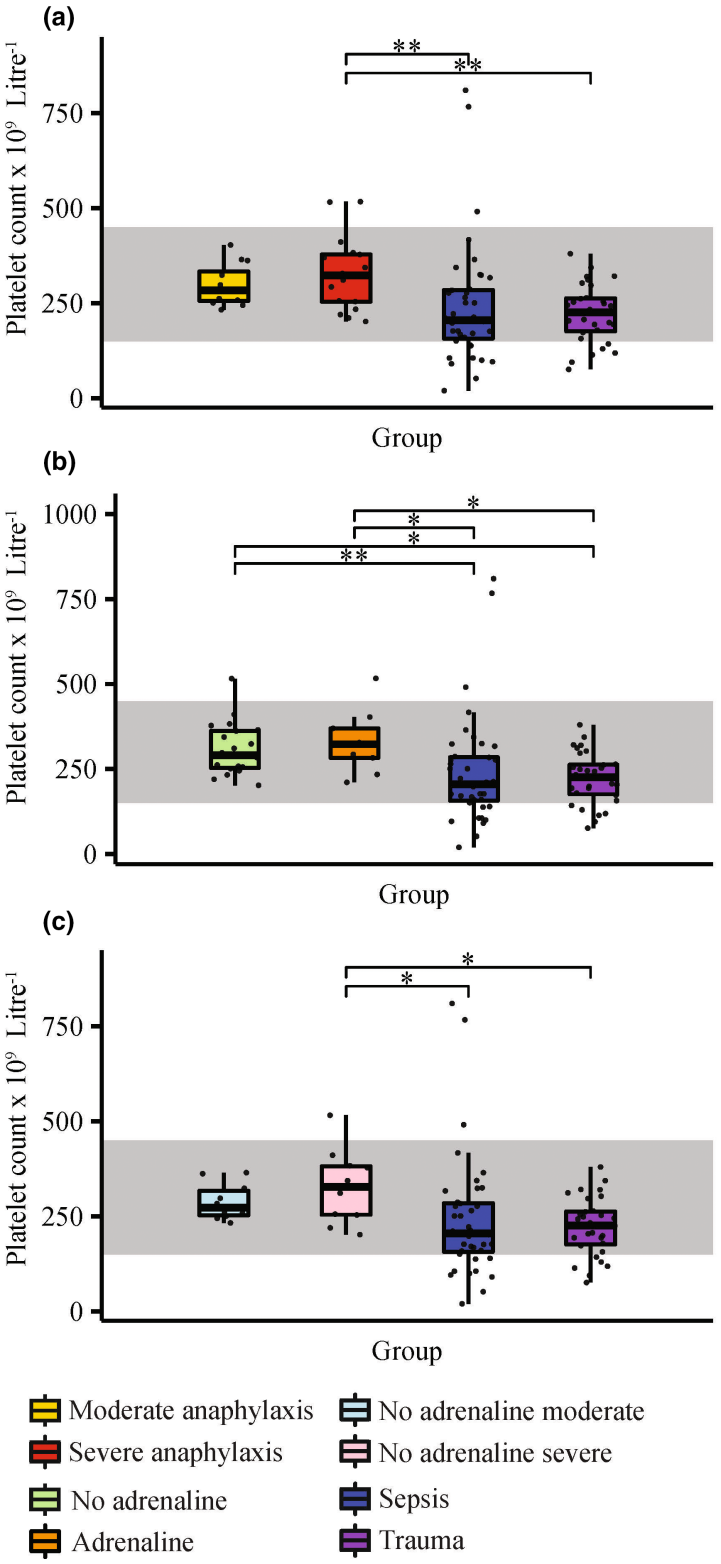
Platelet counts of anaphylaxis, sepsis and trauma patients. Boxplots consist of **(a)** anaphylaxis patients grouped based on severity (severe anaphylaxis *n* = 17, moderate anaphylaxis *n* = 12), **(b)** anaphylaxis patients grouped based on no treatment (*n* = 20) or treatment (*n* = 9) with adrenaline before emergency department arrival and **(c)** anaphylaxis patients who did not receive adrenaline before emergency department arrival grouped based on severity (severe *n* = 10, moderate *n* = 10), and compared with sepsis (*n* = 40) and trauma (*n* = 31) patients. The healthy range of platelet counts is indicated by the grey area. The Kruskal–Wallis test with the Bonferroni adjustment was used to determine statistically significant differences between experiment groups. *adjusted *P‐*value < 0.05, **adjusted *P‐*value < 0.01.

Anaphylaxis patients were initially grouped based on severity and treatment with adrenaline before ED arrival. Overall, platelet counts of anaphylaxis patients were within the healthy range, and sepsis and trauma patients had lowered platelet counts (Figure 6). While within the healthy range, severe anaphylaxis patients had significantly higher platelet counts compared with sepsis (adjusted *P‐*value = 3.61 × 10^−3^) and trauma (adjusted *P‐*value = 6.46 × 10^−3^) patients (Figure 6a). Similarly, patients who did and did not receive adrenaline before ED arrival had platelet counts within the normal range, which were significantly higher compared with sepsis (adjusted *P‐*value = 7.02 × 10^−3^ and adjusted *P‐*value = 2.61 × 10^−2^, respectively) and trauma (adjusted *P‐*value = 1.26 × 10^−2^ and adjusted *P‐*value = 3.46 × 10^−2^, respectively) patients (Figure 6b).

Patients who had not received adrenaline before ED arrival were subsequently grouped based on severity. Sepsis and trauma patients had significantly lower platelet counts than severe no‐adrenaline patients, which were within the normal range (Figure 6c, adjusted *P‐*value = 2.94 × 10^−2^ and adjusted *P‐*value = 4.42 × 10^−2^, respectively).

## Discussion

While our understanding of anaphylaxis is improving, it is still unclear how the reaction progresses, and why severity varies between individuals. We analysed a microarray data set with the aim of identifying pathways and genes dysregulated during acute anaphylaxis, and validated findings. Analysis showed downregulation of platelet aggregation and degranulation in severe anaphylaxis patients, but not sepsis or trauma patients, suggesting this dysregulation is not a general feature of systemic inflammation. Dysregulation of platelet pathways including aggregation and degranulation was seen primarily at T0 in severe anaphylaxis patients who had not received adrenaline before ED arrival. This was confirmed by GSEA, which identified the pathways of platelet aggregation and plug formation, and response to elevated platelet cytosolic Ca^2+^, to be downregulated in severe anaphylaxis. These results suggest the observed suppression of platelet responses occurs early during the reaction.

Platelets are known primarily for their role in thrombosis; however, evidence of their involvement in allergic inflammation is accumulating.^24^, ^25^ Platelets have been shown to contribute towards allergic reactions through release of inflammatory mediators and recruitment of pro‐inflammatory leukocytes.^24^ Platelet‐activating factor (PAF) is released by mast cells in IgE‐mediated reactions, and by basophils in IgG‐mediated reactions, and can result in platelet activation and release of serotonin.^26^ Serotonin release contributes towards reaction severity by increasing vascular permeability.^4^, ^27^ Interestingly, the lungs have been identified as a significant contributor towards platelet biogenesis, accounting for around 50% of platelet production, and platelets have been implicated in numerous allergic lung diseases.^25^, ^28^ A recent study using human IgG receptor‐expressing mouse models found platelet numbers dropped immediately post anaphylaxis.^4^ Platelet activation by human aggregated IgG was demonstrated *in vitro* and *in vivo*, and release of serotonin contributed towards the severity of anaphylaxis.^4^ Depletion of platelets in the mice before the induction of anaphylaxis attenuated the reaction, suggesting platelets are necessary for IgG‐dependent anaphylaxis.^4^ Similarly, in a human cohort with drug‐induced anaphylaxis, platelet activation and consequent reduction in circulating platelets were shown to be associated with reaction severity.^4^ Case series have also demonstrated lowered platelet numbers post anaphylaxis.^29^, ^30^ Combined, this evidence suggests that platelet aggregation and subsequent reduction in circulating platelets is a physiological response to anaphylaxis, which may be linked to downregulation of genes involved in these processes as is observed in this study.

Our validation work demonstrated the downregulation of six platelet‐related genes (*TLN1, GATA1, SELP, SPARC, MPL* and *F13A1*) in anaphylaxis patients compared with healthy controls. Seven genes (*TLN1, GATA1, SELP, GP1BA, MPL, F13A1* and *SPARC*) were also downregulated in patients with severe anaphylaxis who did not receive adrenaline before ED arrival, compared with healthy controls. *TLN1* downregulation was observed in severe and moderate anaphylaxis irrespective of treatment with adrenaline, suggesting downregulation of this gene is independent of treatment and severity. These genes have numerous functions relating to platelet activity, specifically platelet aggregation and activation. Talin‐1, encoded by the *TLN1* gene, is an integrin‐binding cytoplasmic adaptor. Talin is crucial for platelet aggregation and adhesion, and is also involved in T‐cell activation and crucial for T regulatory cell functions.^31^, ^32^, ^33^, ^34^ Specifically, Talin‐deficient mice were shown to have lower numbers of T regulatory cells with reduced function.^34^ Interestingly, T regulatory cell deficiency has been associated with the development of allergic diseases.^35^ No role of *TLN1* in anaphylaxis has previously been described. *GATA1* is a gene known to mediate platelet development.^36^, ^37^ In *in vivo* models of anaphylaxis in mice, reduced *GATA1* activity was shown to heighten IgE‐mast cell‐mediated anaphylaxis and was associated with an amplified T helper 2 cell response.^38^ While the role of *GATA1* in anaphylaxis has only been linked to mast cell activation, reduced *GATA1* activity in anaphylaxis patients may drive reaction severity through downstream effects on platelets and other cell types. *SELP* encodes the P‐selectin protein which mediates platelet‐leukocyte interactions post‐platelet activation, and stabilises platelet aggregates.^39^, ^40^ Interestingly, during IgG‐mediated anaphylaxis in mice, 80% of circulating neutrophils and 90% of circulating monocytes were covered in platelets, although these aggregates did not appear essential for the progression of anaphylaxis.^4^ Platelets bound to leukocytes can release leukocyte activating agents, for example PAF and leukotriene B4, which can activate leukocytes such as neutrophils.^41^ *GP1BA* encodes the glycoprotein Ib‐alpha surface membrane protein, which facilitates platelet adhesion to the endothelium, and can promote platelet activation.^42^ *MPL* encodes the thrombopoietin receptor protein and is important for megakaryocyte proliferation, and subsequently platelet development.^43^ *F13A1* encodes subunits for factor XIII, which helps stabilise clots by supporting platelet adhesion to the endothelium.^44^ Lastly, *SPARC* encodes a glycoprotein secreted by platelets upon activation, which regulates the activity of growth factors including platelet‐derived growth factor.^45^, ^46^

While our work demonstrates for the first time the dysregulation of platelet signatures during acute anaphylaxis in humans, downregulation of platelet pathways has been previously described in severe allergy.^15^ Microarray analysis on patients with profilin‐mediated food allergy showed platelet activation, and aggregation was downregulated in severe allergy compared with moderate and mild allergy.^15^ Specifically, ingenuity pathway analysis found activation of blood platelets, and binding of blood platelets was downregulated in severe allergy patients. Our independent analysis of this data set supported these results (data not shown).

In contrast to results finding downregulation of platelet genes during anaphylaxis, experimental and observational studies have demonstrated the activation of platelets and subsequent aggregation during anaphylaxis.^4^, ^29^, ^30^ We therefore hypothesise that observed downregulation of genes involved in platelet activation and aggregation is a negative feedback response to enhanced platelet activity during anaphylaxis. This negative feedback is initiated early during platelet activation and involves downregulation of platelet adhesive receptors to regulate platelet aggregation, as suggested by the downregulation of *GP1BA*. Regulation of platelet adhesive receptors can occur via four different mechanisms: internalisation, microvesiculation, secretion and ectodomain shedding.^47^ Of particular interest, ectodomain shedding results in rapid downregulation of platelet receptors such as GP1BA, which our analysis showed to be downregulated at the genetic level during severe anaphylaxis. We therefore propose that platelet activation is initiated early during the anaphylactic reaction, followed by rapid downregulation of aggregation receptors to mitigate the response. Interestingly, our work has previously shown the pathway of, ‘cellular response to platelet derived growth factor stimulus’ was upregulated during severe anaphylaxis at ED arrival, further supporting this theory.^19^ Adrenaline is known to promote platelet activation and aggregation, and downregulation of platelet aggregation was not observed in patients who received adrenaline before ED arrival. This could suggest that treatment with adrenaline counteracts the negative feedback response hypothesised.

Study size is a limitation which should be noted. In particular, analysis of subgroups may be underpowered, and further investigations with larger sample sizes are needed to determine whether dysregulation of platelet pathways is dependent on severity and treatment with adrenaline, or universal to all anaphylactic reactions. Additionally, it should be noted that treatment of anaphylaxis patients in the ED depends in part on whether adrenaline was administered before arrival. Specifically, it is uncommon for patients who have had prehospital adrenaline to receive more on ED arrival. These differences in treatment post‐ED arrival may impact T1 gene expression profiles for the adrenaline and no‐adrenaline groups. Bleeding during trauma, and activation of the coagulation cascade, is a potential confounder which likely contributes to low platelet counts seen in this group. Despite limitations, findings from this study support recent literature suggesting an association between platelet activation and aggregation, and severe allergy and anaphylaxis.^4^, ^15^, ^29^, ^30^ Specifically, we have demonstrated for the first time the downregulation of platelet‐related genes during the acute anaphylactic reaction in humans. Our results suggest this involvement occurs early during the acute reaction, and we hypothesise this is a negative feedback response to platelet activation and aggregation. Additionally, our results suggest the downregulation of platelet‐related genes is a unique feature of anaphylaxis, and not a general feature of systemic inflammation. Further understanding pathways and genes driving this platelet involvement would help improve our understanding of the reaction, and potentially guide therapeutic techniques to attenuate the reaction and reduce severity.

## Methods

### Patient cohorts

We analysed two independent patient cohorts: (1) GSE69063 microarray cohort; and (2) validation cohorts (comprising anaphylaxis, sepsis and trauma validation cohorts). Full descriptions of all cohorts can be found below. All patients and healthy controls in the GSE69063 microarray cohort and validation cohorts were recruited through our Critical Illness and Shock Study (CISS), as previously described.^19^, ^48^, ^49^ Anaphylaxis patients in both cohorts were graded using the Brown severity scale by study investigators DF, GA and SB (acknowledged), who were blinded to results.^50^ Severe anaphylaxis was classified by presentation with cardiovascular symptoms including hypotension, cyanosis, loss of consciousness and collapse.

### Gene sets

GSE69063 (McGrath *et al*. 2021)^19^ raw gene expression profile was downloaded from the public functional genomics data repository: Gene Expression Omnibus (GEO). This data set was generated using the Affymetrix Human Gene 2.1 ST Array.

GSE69063 includes samples from severe anaphylaxis (*n* = 12), moderate anaphylaxis (*n* = 6), sepsis (*n* = 20) and previously unpublished trauma (*n* = 11) patients taken during the acute reaction; and controls age and sex matched to the anaphylaxis (*n* = 10), sepsis (*n* = 11) and trauma (*n* = 12) cohorts. Samples were taken at presentation to the ED (T0), and 1 h later (T1). Triggering allergens included foods and drugs. Clinical characteristics of all cohorts are described in Table 1.

### Microarray analysis

Microarray data were analysed in the R environment for statistical computing (version 4.0.3), as previously described.^19^ Briefly, this included robust multi‐array expression (RMA) measure normalisation using the *oligo* Bioconductor package, and identification of differentially expressed genes (DEGs; false discovery rate (FDR) adjusted *P*‐value < 0.05 and a log_2_ fold change ≥ 0.6, or ≤−0.6) using the *limma* Bioconductor package.^51^ Probe sets were mapped to genes using hugene21sttranscriptcluster (version 8.7.0). DAVID Bioinformatics Resource (version 6.8) and GSEA were used for pathway analysis.

### Validation: RNA Extraction and quality control

Samples taken at T0 from an independent cohort of 31 anaphylaxis patients (12 severe) and 10 age‐ and sex‐matched healthy controls were selected to validate findings from the GSE69063 microarray. Total RNA was extracted employing the PAXgene Blood RNA Extraction Kits (PreAnalytiX GmbH, Hombrechtikon, Switzerland) using an automated QIAcube protocol (QIAGEN, Melbourne, Australia), following the manufacturer's recommendations. RNA quantity was measured using the Qubit Flex Fluorometer 4 Extended Range (XR) assay (ThermoFisher Scientific, Melbourne, Australia), and RNA integrity number (RIN) was measured using a Bioanalyzer (Agilent Technologies, Santa Clara, United States). Median RNA quantity was 444 ng μL^−1^ (IQR 380–503 ng μL^−1^). Median RIN was 8.9 (IQR 8.6–9.1).

### qRT‐PCR analysis

Triplicate quantitative reverse transcription‐polymerase chain reaction (qRT‐PCR) reactions were performed using TaqMan gene expression assay probes, on a ViiA™7 Real‐Time PCR System (ThermoFisher Scientific, Melbourne, Australia). No template controls, no reverse transcription controls and water blanks were run for each target. Expression data were normalised to four housekeeper genes (*PSMC4*, *ELF1*, *POP4* and *ATP6*), selected following analysis of samples using TaqMan™ Array Human Endogenous Control plates, employing GenEx software. The 2^−ΔΔCT^ method was applied to determine normalised relative expression values.

### Statistical analysis

To determine statistically significant differences in gene expression between anaphylaxis patients and healthy controls, Welch's unequal variance *t*‐tests were used. Anaphylaxis patients were subsequently grouped based on severity or treatment before ED arrival, and one‐way ANOVA with Tukey's honestly significant difference (HSD) adjustment was used to determine statistically significant differences between normally distributed patient groups. In cases of skewed data, the Kruskal–Wallis test with the Bonferroni adjustment was used to determine statistically significant differences between groups.

### AUTHOR CONTRIBUTIONS

All authors were involved in editing, review and approval of the final manuscript. FM contributed towards writing of the original manuscript draft, performed laboratory experiments, data analyses and data interpretation. AF contributed towards study concept and design and data curation. DF contributed towards study design and concept. SM contributed towards data curation and investigation. GA contributed towards study design, data curation and investigation. DF, SM and GA contributed towards participant recruitment. AW contributed towards data analyses and interpretation. EB contributed towards study concept, data curation, study design and interpretation of data.

## Conflict of interest

The authors declare they have no conflicts of interest to disclose.

## Supporting information


**Supplementary figure 1**.
Supplementary figure 2.
Supplementary figure 3.

## Data Availability

Microarray data from which the findings of this study were generated are publicly available in the Gene Expression Omnibus at https://www.ncbi.nlm.nih.gov/geo/query/acc.cgi?token=wpenmkucldaxtcr&acc=GSE69063, reference number GSE69063. Other data pertaining to this study can be made available upon request.
