# Supplementary material for: Genes involved in platelet aggregation and activation are downregulated during acute anaphylaxis in humans
Source: Clin Transl Immunology. 2022 Dec 26;11(12):e1435. doi: 10.1002/cti2.1435 (PMC9791329; doi:10.1002/cti2.1435)
Supplement: Supplementary file 1 — Supplementary figure 1. Supplementary figure 2. Supplementary figure 3. [file CTI2-11-e1435-s001.pdf]

Supporting Information

Francesca McGrath[1], Abbie Francis[1,2], Daniel Fatovich[1,3,4], Stephen Macdonald[1,3,4], Glenn Arendts[1,3,5], Andrew J Woo[6,7], Erika Bosio[1,3,4].

[1]Centre for Clinical Research in Emergency Medicine, Harry Perkins Institute of Medical Research, Perth, WA, Australia

[2]Telethon Kids Institute, Centre for Child Health Research, The University of Western Australia, Nedlands, WA, Australia

[3]Division of Emergency Medicine, Medical School, University of Western Australia, Perth, WA, Australia

[4]Emergency Department, Royal Perth Hospital, Perth, WA, Australia

[5]Emergency Department, Fiona Stanley Hospital, Perth, WA, Australia

[6]Laboratory for Cancer Medicine, Harry Perkins Institute of Medical Research, Perth, WA, Australia

[7]School of Medical and Health Sciences, Edith Cowan University, Perth, WA, Australia

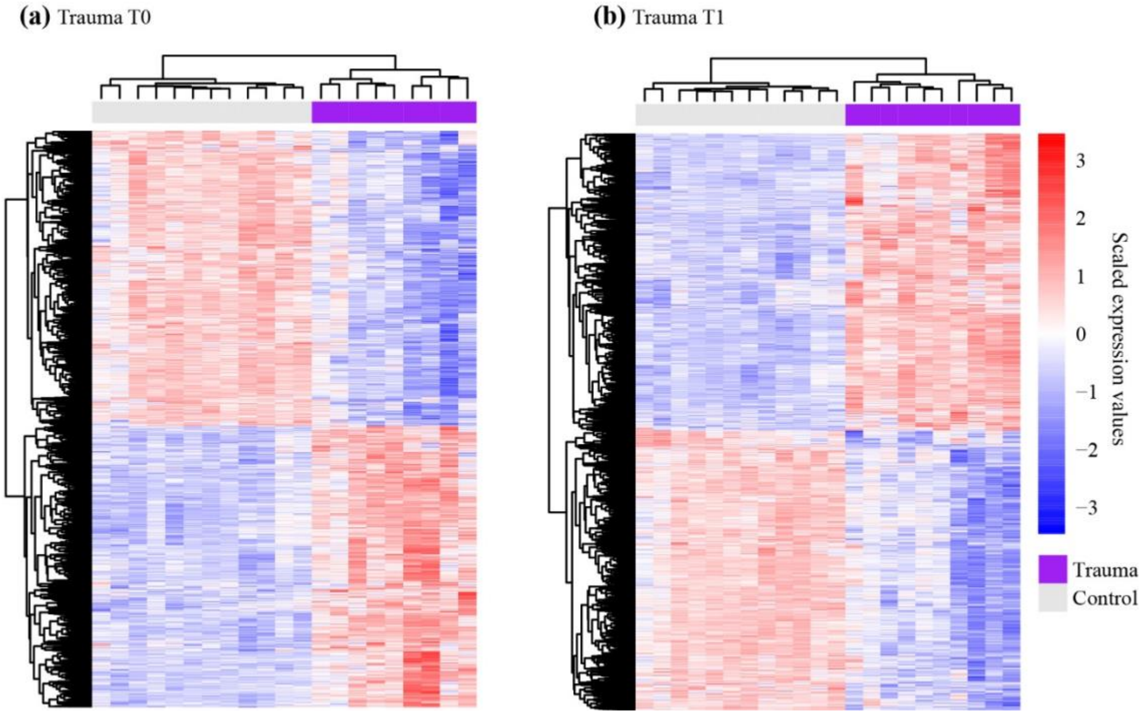

Supplementary figure 1: Heatmaps of differentially expressed genes identified in the microarray analysis (gene set GSE69063) of trauma patients (n = 11) compared to matched controls (n = 12). (a) The 1790 differentially expressed genes identified at T0. **(b)** The 2333 differentially expressed genes identified at T1. Heatmaps were generated in R, with scaled expression values represented as a colour spectrum from low (blue) to high (red). Relationship between genes and patients are represented along the top (samples) and left (genes) borders.

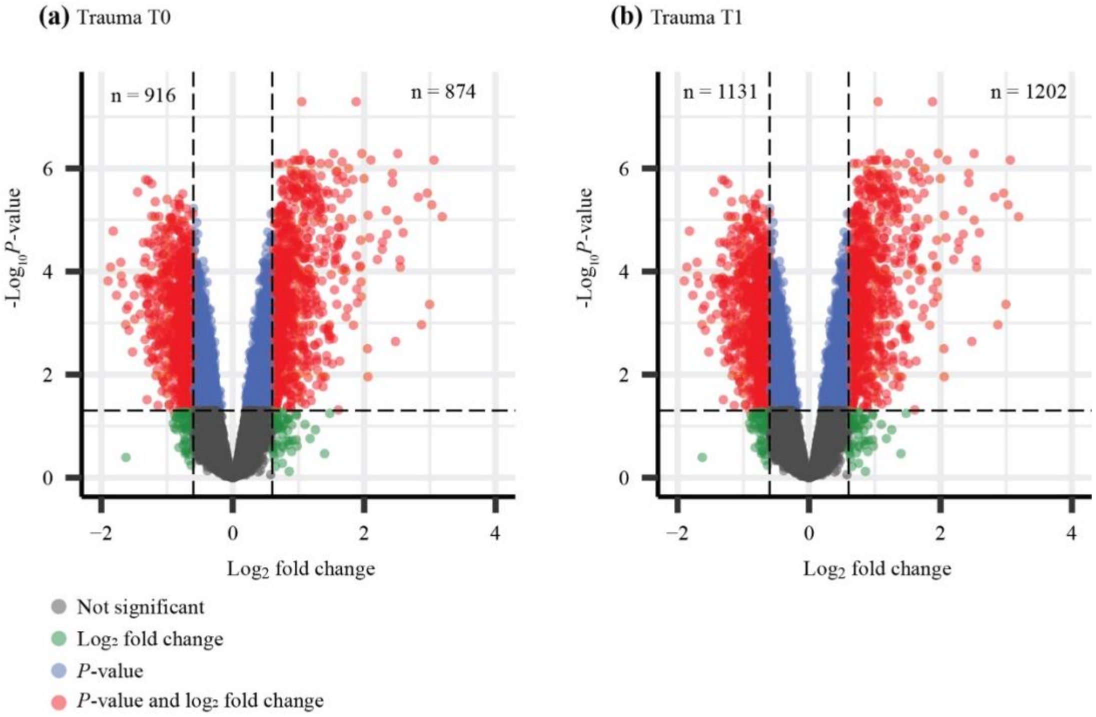

Supplementary figure 2: Volcano plots highlighting significant genes, identified through microarray analysis (gene set GSE69063) at (a) T0 and (b) T1 in trauma patients (n = 11) compared to matched healthy controls (n = 12). Volcano plots were generated in R. Genes were identified as: not significant (grey circles), significant Log_2 fold change (blue circles), significant adjusted *P*-value (green circles), or significant adjusted *P*-value and Log_2 fold change (red circles).

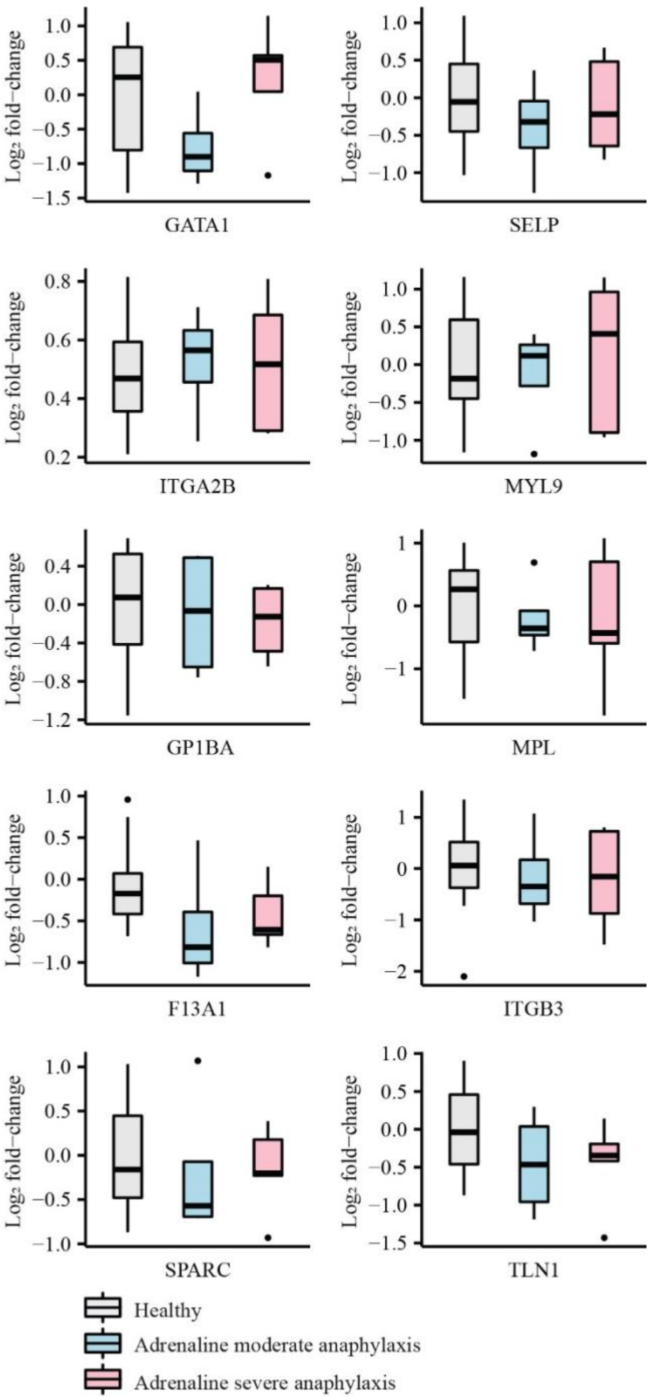

Supplementary figure 3: qRT-PCR validation of platelet-related validation panel genes. Log_2 fold-changes were determined using the $2^{-\Delta\Delta CT}$ method where groups were normalised to healthy controls. Comparison groups included anaphylaxis patients who received adrenaline before ED arrival (n = 9), grouped as severe (n = 5), or moderate (n = 4), and healthy controls (n = 10). One-way ANOVA with the Tukey honesty significance difference (HSD) adjustment was used to determine statistically significant differences between experiment groups.
